# A muscular dystrophy associated with bi‐allelic *LEMD2* variants: Expanding the genotype of nuclear envelopathies

**DOI:** 10.1111/bpa.70082

**Published:** 2026-03-03

**Authors:** Marc Pauper, Heike Kölbel, Iakowos Karakesisoglou, Anne Schänzer, Johann Böhm, Rachel Thompson, Nicolai Kohlschmidt, Bernd Ringel, Gisèle Bonne, Katja Neuhoff, Andrea Gangfuß, Ozge Aksel Kilicarslan, Sergi Beltran Agullo, Andreas Hentschel, Ulrike Schara‐Schmidt, Hanns Lochmüller, Kiran Polavarapu, Andreas Roos

**Affiliations:** ^1^ Centro Nacional de Análisis Genómico (CNAG) Barcelona Spain; ^2^ Universitat de Barcelona (UB) Barcelona Spain; ^3^ Department of Pediatric Neurology Centre for Neuromuscular Disorders, University Duisburg‐Essen Essen Germany; ^4^ Department of Biosciences Durham University Durham UK; ^5^ Institute of Neuropathology, Justus Liebig University Giessen Giessen Germany; ^6^ Institut de Génétique et de Biologie Moléculaire et Cellulaire (IGBMC), Inserm U1258, CNRS UMR7104 Université de Strasbourg Illkirch France; ^7^ Children's Hospital of Eastern Ontario Research Institute Ottawa Ontario Canada; ^8^ Institute of Clinical Genetics and Tumour Genetics Bonn Bonn Germany; ^9^ National Center of Genetics (NCG) Dudelange Luxembourg; ^10^ Sorbonne University INSERM, Institut de Myologie, Centre de Recherche en Myologie Paris France; ^11^ Departament de Genètica Microbiologia i Estadística, Facultat de Biologia, Universitat de Barcelona (UB) Barcelona Spain; ^12^ Leibniz‐Institut für Analytische Wissenschaften‐ISAS‐e.V Dortmund Germany; ^13^ Division of Neurology, Department of Medicine The Ottawa Hospital Ottawa Ontario Canada; ^14^ Brain and Mind Research Institute, University of Ottawa Ottawa Ontario Canada; ^15^ Department of Neurology Medical Faculty and University Hospital Düsseldorf, Heinrich Heine University Düsseldorf Germany

**Keywords:** muscle proteomics, muscular dystrophy, NMD‐GPS, nuclear envelope, proteogenomics

## Abstract

Proteomics‐guided exome re‐analysis identifies bi‐allelic variants in the nuclear envelope LEMD2 gene, expanding its phenotypic spectrum. Created in BioRender. Pauper, M. (2026) https://BioRender.com/xamvo92.

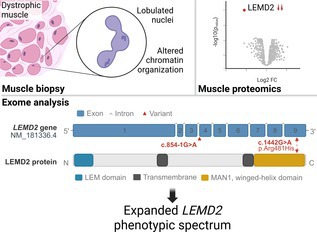

Defects in genes encoding nuclear envelope‐related proteins can lead to distinct neurological phenotypes, even when the same gene is affected [1]. For example, *MATR3* variants can cause either adult‐onset distal myopathy or rare forms of amyotrophic lateral sclerosis (ALS) [2]. Similarly, *SYNE1* mutations have been linked to arthrogryposis multiplex congenita type 3, Emery–Dreifuss muscular dystrophy (EDMD) type 4, and spinocerebellar ataxia type 8 [2]. *SYNE2* variants may cause EDMD type 5, as well as neurodevelopmental conditions like autism and intellectual disability [2]. *LMNA* variants are associated with a range of disorders including myopathies, neuropathies, partial lipodystrophies, and progeroid syndromes [2]. On the ultrastructural level, these genetic defects often lead to disintegration of the inner and outer nuclear membrane, perturbed chromatin distribution, or both.

LEMD2, a nuclear envelope‐associated protein, plays a role in nuclear structure, integrity, and reformation after mitosis. It also acts as a chromatin binding mediator [3] *LEMD2* variants have been linked to arrhythmic cardiomyopathy, juvenile‐onset cataract (MIM:212500) with sudden cardiac death [4] and Marbach‐Rustad progeroid syndrome (MIM:619322) [5] Mild muscle weakness has been reported in some cases, but no association with profound myopathy has been described until now.

We report a paediatric patient with syndromic presentation including congenital myopathy, mild intellectual disability, and respiratory dysfunction. Muscle proteomics revealed a significant decrease of LEMD2 protein abundance. Exome re‐analysis was prompted by this proteomic finding and uncovered biallelic *LEMD2* variants: c.854‐1G>A (splice site) and c.1442G>A (p.Arg481His). In silico modelling evaluated the structural impact of the p.Arg481His substitution. Microscopic studies revealed altered distribution of nuclear envelope‐related proteins.

## 1 MATERIALS AND METHODS

See Supporting Information Document S1.

### 1.1 Ethics approval

The institutional review board of the University Medicine Essen (Germany) approved the NMD‐GPS study (19‐9011‐BO), a research initiative aiming at diagnosing and understanding neuromuscular disorders through comprehensive genomic, proteomic, phenotypic, and clinical data integration (https://nmd-gps.net/). Patient and parents gave informed consent to participate in the study before taking part.

## 2 RESULTS

### 2.1 Clinical and microscopic biopsy findings

**FIGURE 1:**
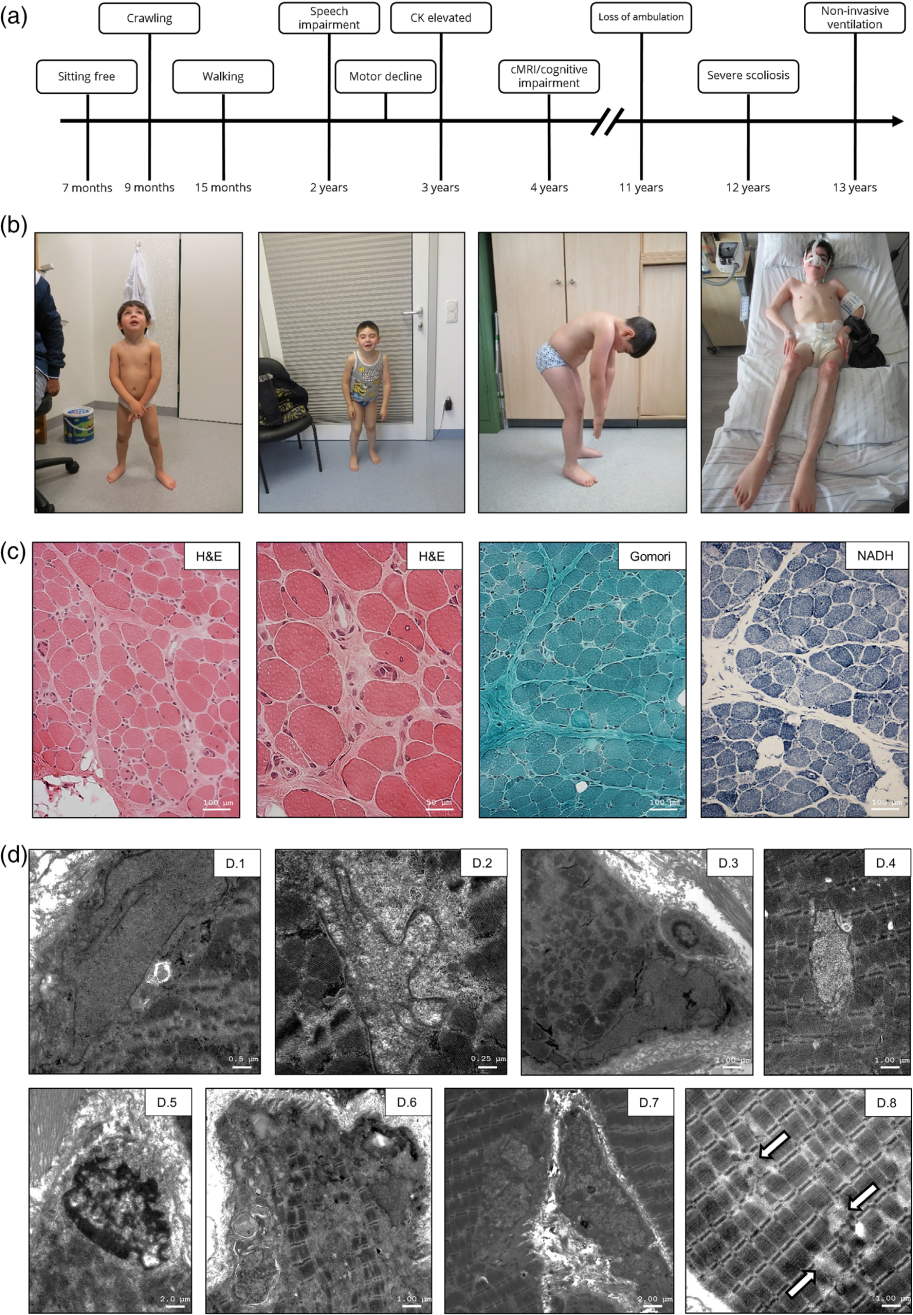
Clinical findings: (A) Chronological sequence of the patient's developmental, diagnostic, therapeutic, and clinical events. (B) Photographs of the patient at the age of 4, 6, 8 and 13 years showing the development of elbow contractures as a first sign, a rigid spine at the age of 8 years and at 14 years loss of ambulation and respiratory insufficiency. (C) Histological findings in the quadriceps biopsy of our patient include notable endomysial and perimysial fibrosis, increased adipose tissue, marked fibre size variation and numerous atrophic fibres (H&E and Gomori‐stains). Centrally located myonuclei observed in <3% of fibres (H&E stain). No signs of mitochondrial vulnerability based on Gomori‐ and NADH‐TR stains. (D) Ultrastructural findings reveal myonuclei of abnormal shape located both at the periphery and centre of myofibres (D.1–D.7). Some myonuclei and nuclei of non‐muscle cells show nuclear splitting (segmentation), indicative of a pathological phenotype (D.6 and D.7). The myofibrillar architecture is focally loosened and partially disrupted (white arrows), with no specific sarcomeric pathology present (small arrows).

We report a 13‐year‐old boy (non‐consanguineous family) with a progressive neuromuscular disorder and a history of an older sibling who died at age seven with a suspected muscular dystrophy. Early motor and speech delays were noted, followed by progressive muscle weakness, elevated CK levels and loss of ambulation at the age of 11 years. Cognitive testing revealed mild impairment. The patient developed joint contractures, scoliosis requiring spinal fusion, and restrictive respiratory failure managed with nocturnal ventilation. Despite significant neuromuscular symptoms, cardiomyopathy has not developed. Growth remains impaired, with endocrine testing showing low growth hormone (GH)‐dependent factors, though GH therapy was declined by the family. Clinical history is illustrated in Figure 1A and photos of the patient are shown in Figure 1B.

Muscle biopsy sections demonstrated notable endomysial and perimysial fibrosis, along with increased adipose tissue. Muscle fibres were polygonal to mildly rounded, with marked fibre size variation and numerous atrophic and some small regenerating fibres (Figure 1C). Myonuclei were predominantly peripherally located, with centrally located nuclei observed in <3% of fibres. Occasional necrotic fibres with evidence of phagocytosis were seen. No signs of mitochondrial vulnerability were observed. Ultrastructural analysis revealed abnormally lobulated myonuclei (>10%), some of which exhibited irregular chromatin distribution and occasional subtle disruption of sarcomeric architecture (Figure 1D).

### 2.2 Proteomic findings

**FIGURE 2:**
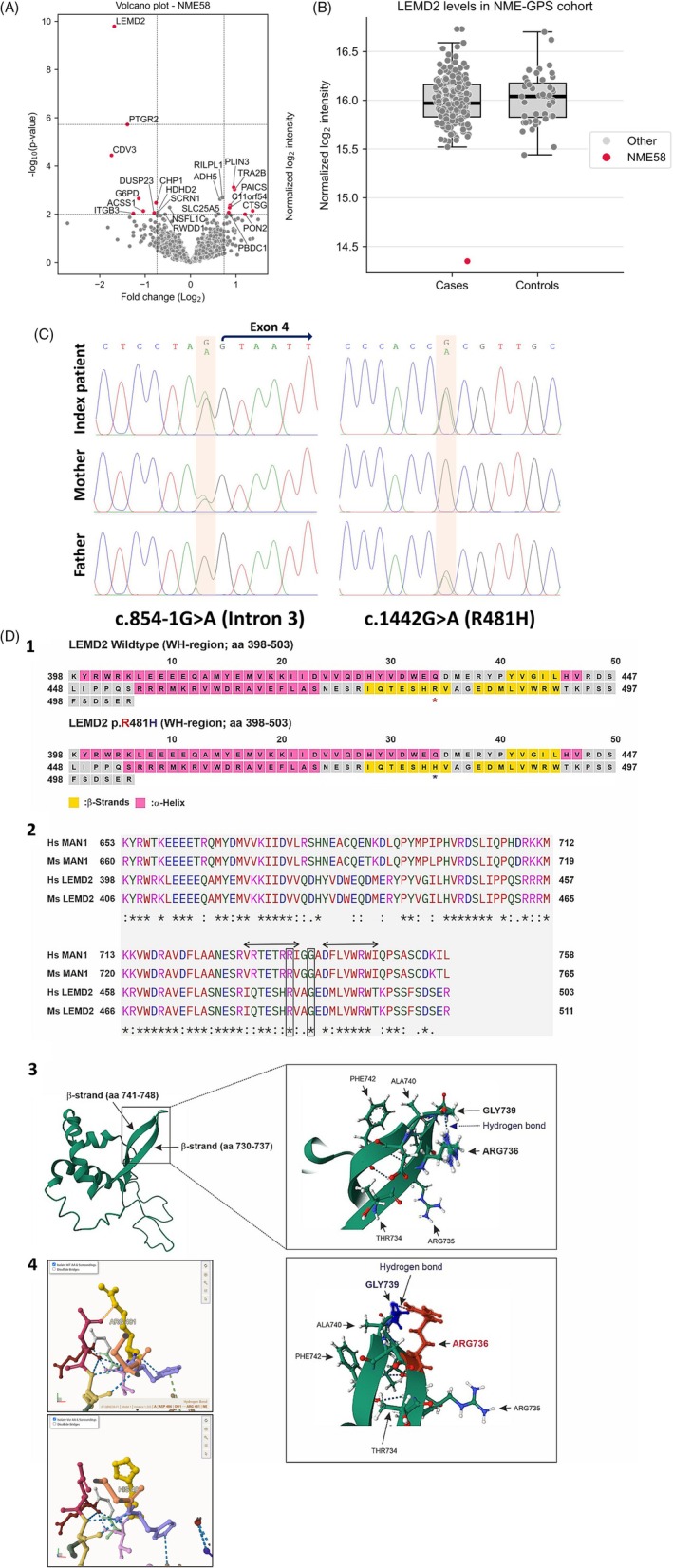
Molecular findings: (A) Volcano plot based on proteomic signature of quadriceps muscle of the index patient highlighting the massive statistically significant decrease of LEMD2. (B) Boxplots showing LEMD2 abundance in muscle of all patients of the NMD‐GPS cohort as well as healthy controls. NME58 corresponds to the index patient. (C) Sanger sequencing results illustrating segregation of the compound heterozygous *LEMD2* variant. (D) (1) Secondary structure predictions for wild‐type and p.Arg481His mutant LEMD2 peptides (aa 398–503, WH domain) using PSIPRED showed no major structural differences. (2) BLAST alignment of human LEMD2 WH with human MAN1 (*LEMD3*) revealed 56% identity and 75% similarity. Clustal Omega alignment with human/mouse LEMD2 and MAN1 confirmed conservation within this domain. (3) In the MAN1 NMR structure (PDB: 2CH0), R736 within a β‐strand forms a stabilizing hydrogen bond with G739 in wing1. These align with Arg481 (R481) and Gly484 (G484) in LEMD2, suggesting the p.Arg481His variant may disrupt this bond and destabilize the WH domain. Given WH domain conservation, the mutation could impair domain folding or function. (4) Miztli/Missense3D analysis predicted a loss of hydrogen bond between Arg481 and Asp486.

Mass spectrometry‐based proteomics data processing of 216 individuals of the NMD‐GPS cohort resulted in a total of 5181 proteins identified across the entire cohort, of which 1778 were identified in at least 90% of participants. In the patient, after quality filtering, 3006 proteins were available for further analyses. Statistical testing resulted in 22 outliers that were nominally significant at an alpha level of 0.01, of which 12 were downregulated and 10 were upregulated (Table S1). LEMD2, with a fold change (FC) of 0.31 (log_2_FC = −1.68), was the only outlier protein reaching statistical significance after multiple‐testing correction (*p*‐value = 1.618 × 10^−6^, adjusted *p*‐value = 4.177 × 10^−6^, Figure 2A,B). Throughout the cohort, LEMD2 had a very low missing rate (1.85%) and its identification and quantification were supported by 14 precursor ions.

### 2.3 Genetic and in silico findings

Exome sequencing revealed compound heterozygous *LEMD2* (NM_181336.4) variants: c.854‐1G>A (affecting the intron 3 splice acceptor site) and c.1442G>A (p.Arg481His) in exon 9. A list of variants identified in other neuromuscular‐related genes is provided in Table S2. Sanger sequencing including parental DNA samples confirmed segregation by showing that the c.1442G>A variant was inherited from the father while the c.854‐1G>A variant was inherited from the mother (Figure 2C).

Variant c.854‐1G>A is predicted to abolish the splice‐acceptor site (SpliceAI score = 1.0) and is highly deleterious per CADD (Phred score = 34, GRCh38‐v1.7). It is absent from gnomAD (v4.1.0) and the RD‐Connect GPAP database and unreported in ClinVar and the medical literature, confirming its extreme rarity across all populations. Variant c.1442G>A (p.Arg481His) is predicted to be deleterious by CADD (Phred score = 27, GRCh38‐v1.7) and AlphaMissense (score = 0.8). It is extremely rare (allele frequency: 1.239 × 10^−6^), with only two heterozygotes reported in gnomAD (v4.1.0). Both variants are classified as of uncertain significance (VUS) under ACMG criteria.

To evaluate the structural consequences of the LEMD2 Arg481 mutated residue, complementary approaches were employed. Secondary structure predictions for wild‐type and p.Arg481His mutant LEMD2 peptides (aa 398–503, WH domain) using Phyre2 (v2.2) and PSIPRED showed no major structural differences. Based on MAN1 (*LEMD3*)‐derived homology modelling, Arg481 of LEMD2 is predicted to interact with Gly484, while MIZTLI‐based structural refinement suggests an alternate hydrogen bond with Asp486. Both models highlight the potential role of Arg481 in local structural stabilization (Figure 2D). Dynamut2 predicted that the Arg481His substitution is destabilizing for the protein structure (ΔΔ*G* = −1.67 kcal/mol).

### 2.4 Immunofluorescence findings

Immunofluorescence analyses focusing on the distribution of nuclear envelope‐related proteins revealed normal localization of the nuclear pore protein KPNB1/Importin subunit beta‐1, while occasional myonuclei showed nucleoplasmic immunoreactivity of Emerin, Lamin A/C, and Lamin B1. For Matrin‐3, immunofluorescence analysis occasionally identified myonuclei with an irregular distribution pattern, including perinuclear immunoreactivity. These immunofluorescence findings are summarized in Figure S1.

## 3 DISCUSSION

This study describes a patient with an Emery–Dreifuss muscular dystrophy (EDMD)‐like muscle phenotype without cardiomyopathy, where initial whole‐exome sequencing failed to identify a definitive genetic cause. Proteomics revealed significantly reduced LEMD2 levels in the patient compared to controls and a neuromuscular disease cohort. Re‐analysis of the exome data guided by these findings uncovered two previously unreported, likely pathogenic variants in the *LEMD2* gene. LEMD2 is essential for maintaining nuclear architecture, organizing chromatin, and facilitating nuclear envelope reassembly. Structural alterations in LEMD2 have been linked to several human disorders, including autosomal dominant Marbach–Rustad progeroid syndrome (MIM:619322) and autosomal recessive juvenile‐onset cataract‐46 with or without arrhythmic cardiomyopathy (MIM:212500) [4]. Here we report a novel association of LEMD2 with an EDMD‐like clinical muscle phenotype. Muscle biopsy from this patient displayed centralized and abnormal lobulated as well as segmented nuclei. From the ultra‐structural perspective, these observed ultrastructural changes are more consistent with a general dystrophic histomorphological phenotype rather than a specific EDMD‐like nuclear pathology [6]. Immunofluorescence further confirmed perturbations in nuclear envelope‐associated proteins, supporting the notion that *LEMD2* dysfunction contributes to nuclear instability. A nearby dominant missense variant, p.Ser479Phe, has been linked to Marbach‐Rustad progeroid syndrome (MIM:619322), with progeria‐like features and neurologic deficits, underscoring the gene's pleiotropic impact [5]. Pleiotropic phenotypes have also been described in other nuclear envelopathies, for example, in LMNA‐related phenotypes [7]. Finally, our patient's mild intellectual disability, along with prior evidence linking *LEMD2*‐regulated genes to neurodevelopmental disorders, suggests that chromatin abnormalities resulting from nuclear envelope disruption may also impair cognitive function [8].

Although nuclear irregularities were consistently observed in our patient's muscle biopsy, it is important to note that similar alterations—such as nuclear enlargement, lobulation, chromatin condensation changes, and peripheral displacement—have been described not only in disorders caused by mutations affecting nuclear envelope proteins, but also in other neuromuscular conditions, including congenital myopathies caused by ACTA1 defects [9].

Taken together, these findings (i) expand the phenotypic spectrum associated with recessive *LEMD2* mutations, (ii) highlight *LEMD2*'s central role in nuclear architecture and its involvement in muscle pathology, supporting its inclusion among nuclear envelope‐related disease genes, and (iii) underscore the value of proteogenomic approaches at the omics intersection in diagnosing rare and complex neuromuscular disorders.

## AUTHOR CONTRIBUTIONS

Ulrike Schara‐Schmidt, Hanns Lochmüller, Kiran Polavarapu and Andreas Roos designed the study. Heike Kölbel, Andrea Gangfuß and Ulrike Schara‐Schmidt provided clinical data. Nicolai Kohlschmidt and Johann Böhm performed genetic testing. Andreas Hentschel, Katja Neuhoff and Bernd Ringel performed biochemical laboratory work. Anne Schänzer conducted microscopic studies. Marc Pauper, Sergi Beltran Agullo, Andreas Roos, Ozge Aksel Kilicarslan, Rachel Thompson, Hanns Lochmüller, and Kiran Polavarapu analysed omics data and performed data intersection. Marc Pauper, Kiran Polavarapu and Iakowos Karakesisoglou performed in silico protein domain analysis. Marc Pauper and Andreas Roos drafted the manuscript. Gisèle Bonne provided critical feedback to the manuscript.

## CONFLICT OF INTEREST STATEMENT

The authors declare no conflicts of interest.

## Supporting information


**Document S1.** Overview of applied methods and materials used: A detailed description of genetic testing, in silico protein modelling, microscopic investigations, proteomics, and immunofluorescence studies is provided, along with a list of the materials used, offering clarity on the technologies and tools utilized.

**Figure S1.** Immunofluorescence‐based study of nuclear envelope proteins in *LEMD2* patient and control muscle: Fluorescence‐based immunolabelling of five different nuclear envelope‐related proteins, KPNB1/Importin subunit beta‐1, Emerin, Lamin A/C, Lamin B1 & Matrin‐3 on 7 μm quadriceps biopsy sections of an age matched non‐disease control (left column) and the *LEMD2* patient (right column). Altered immunoreactivity for the respective proteins is indicted by white arrows and in sum indicated a pathophysiological impact of the compound heterozygous *LEMD2* variants on distribution of other nuclear envelope resident proteins.

**Table S1.** Outlier proteins in LEMD2 patient.

**Table S2.** Overview of all genetic variants identified in neuromuscular genes in the patient.

## Data Availability

The data that support the findings of this study are openly available in ProteomeXchange at https://www.proteomexchange.org/, reference number PXD065567. Genomic data are available to registered users of the RD‐Connect Genome‐Phenome Analysis platform (GPAP) at platform.rd‐connect.eu.
