# Supplementary material for: A muscular dystrophy associated with bi‐allelic LEMD2 variants: Expanding the genotype of nuclear envelopathies
Source: Brain Pathol. 2026 Mar 3;36(4):e70082. doi: 10.1111/bpa.70082 (PMC13239970; doi:10.1111/bpa.70082)
Supplement: Supplementary file 1 — Document S1. Overview of applied methods and materials used: A detailed description of genetic testing, in silico protein modelling, microscopic investigations, proteomics, and immunofluorescence studies is provided, along with a list of the materials used, offering clarity on the technologies and tools utilized. [file BPA-36-e70082-s004.docx]

**Genetic analysis**

Patient underwent whole exome sequencing using custom Twist_HumanRefSeqPanel_37Mb. Exome data (fastq files) from the patient was processed at the Centro Nacional de Análisis Genómico (CNAG), Barcelona, Spain, and variant analysis was carried out using the RD-Connect Genome-Phenome Analysis Platform (GPAP) (https://platform.rd-connect.eu).^1^ Initial analysis was performed against detailed phenotypic data uploaded to GPAP and using a virtual neuromuscular gene panel obtained from the muscle gene table (<https://www.musclegenetable.fr/>).^2^ The variant analysis was expanded further to all known disease-causing genes from OMIM database (<https://omim.org/>). Variants in high or moderate impact were further filtered using gnomAD MAF (minor allele frequency) <0.001 and GPAP internal frequency <0.01. Variant interpretation along with *in silico* predictions were assessed using Franklin (<https://franklin.genoox.com/clinical-db/home>) and Mobidetails (<https://mobidetails.chu-montpellier.fr/>).^3^

Segregation analysis was performed by Sanger sequencing of *LEMD2* exon 4 (forward 5’-CTCCTTATTAGAGCTGATGGC-3’; reverse 5’-CAGGGCTTGAAAGACCAATAGG-3’) and exon 9 (forward 5’-AGAGCCTGGAAATGTGTGCC-3’; reverse 5’-CAAGTGTGAATTCAGCACCGC-3’) including adjacent splice-relevant regions of the index patient and both healthy parents. Mutations were numbered according to GenBank NM_181336.4 and NP_851853.1 with +1 corresponding to the A of the ATG translation initiation codon.

***In silico* protein domain analysis**

Secondary structure predictions for the wild-type and p.R481H mutant LEMD2 protein were generated using peptide sequences corresponding to the Winged Helix (WH) region of human LEMD2 (residues 398–503; NP_851853.1), with Phyre2 (v2.2) and PSIPRED.^4^ As both tools yielded identical results, only the PSIPRED predictions are shown (Figure 2D1).

For sequence alignment of the Winged Helix (WH) region on human (Hs) LEMD2 with orthologous and related protein sequences (Figure 2D2), the WH region (residues 398–503 in human LEMD2; NP_851853.1) was aligned using Clustal Omega with the corresponding region from mouse (Ms) LEMD2 (NP_001074662.2) and with human and murine MAN1 (LEMD3) sequences (Hs NP_055134.2; Ms NP_001074662.2). Conserved (*) and similar (: or .) residues were highlighted in the alignment.

The structures shown in Figure 2D3 are based on the solution NMR structure of the human MAN1 C-terminal domain (residues 655–775), which was obtained from the Protein Data Bank (www.rcsb.org; PDB ID: 2CH0).^5^ The MAN1 structure was visualized using the MOL* viewer. Amino acid (aa) residues were numbered according to the full-length human MAN1 protein (UniProt: Q9Y2U8). The twisted antiparallel b-sheet strands of the carboxy-terminal half of MAN1 were highlighted, with the encompassing amino acid residues indicated in parentheses. The short loop (wing1) connecting the antiparallel b-strands includes residues GLY738, GLY739 and ALA740. Key amino acid residues involved in the hydrogen bond (dashed line)—between the ARG736 side chain and GLY739—were labelled and color-coded in red and blue, respectively, to highlight their positions and interactions (Figure 2D3, lower panel).

Additionally, *in silico* analysis for structural impact of the missense variant *LEMD2*: p.Arg481His were done using the Alphafold predicted structure AF-Q8NC56-F1-v4.^6^ For structural analysis of the missense variant, we used MIZTLI 3D engine (https://biokerden.eu/) to compare the wild type and mutant structures of LEMD2 protein. Structural and folding energy changes (ΔΔG) between wild type and mutant LEMD2 using Missense3D and Dynamut2.^7,8^

**Mass spectrometry and data analysis**

Triplicate proteomics data of patient and 215 additional samples of the NMD-GPS cohort were prepared and analyzed as described in Pauper and colleagues.^9^ Leveraging this larger proteomic dataset enhanced the depth and robustness of protein identification and quantification. To process the log_2_-transformed triplicate intensity measurements, a conditional approach was applied based on the number of non-missing replicates values and their coefficient of variation (CV):

*Only one replicate value:*

- Condition: When only one replicate value out of three was available.
- Action: Value excluded and protein intensity considered missing in the individual.

*Low CV or two replicate values:*

- Condition: When all three replicate intensity values are available, and their CV was below 10%, or when only two replicates were available.
- Action: The mean of the intensity values was calculated directly.

*High CV and three replicate values:*

- Condition: When all three replicate intensity values are available, but the CV is equal to or exceeds 10%, suggesting significant variability.
- Action: A modified Z-score method utilizing the median absolute deviation was calculated for each replicate intensity, according to the following formula:

$$Z_{i}=\frac{\left| \left( X_{i}-median\left( X \right) \right) \right|}{median\left( \left| \left( X-median\left( X \right) \right) \right| \right)}$$

Intensity values with a modified Z-score exceeding a threshold of *m=*2 were identified as outliers and excluded. With three replicate values and a threshold of *m=2*, this method removes the value that deviates most from the median if its deviation is at least twice that of the value closest to the median. The mean of the remaining intensity values was then computed.

Protein outliers were identified using PROTRIDER, an adaptation of the OUTRIDER algorithm.^10,11^ PROTRIDER is designed to detect outliers within a population, making use of an autoencoder to automatically control for confounders in the data, and applying a statistical test on the observed and expected protein intensities using two-sided Gaussian p-values. Multiple testing correction is then applied with the method of Benjamini and Yekutieli.^12^ Proteins missing in 80% or more of participants were excluded from PROTRIDER analysis.

**Proteomics-based candidate pathogenic variant detection**

An in-house developed python tool was used to extract rare genomic variants (gnomAD and RD-Connect GPAP internal allele frequency < 0.01) detected in the sequence of genes encoding protein expression outliers (PROTRIDER p-value < 0.05). These rare variants were filtered to include only variants with a predicted moderate or high impact on protein product (VEP predicted impact).^13^ Furthermore, variants known to be (likely) benign in the ClinVar database were excluded.^14^

**Electron Microscopy**

Electron microscopic studies of ultrathin sections of glutaraldehyde-fixed, Epon-embedded, transversely and longitudinally oriented skeletal muscle samples were performed as described before by Malaichamy *et al*.^15^

**Immunofluorescence studies**

Fluorescence-based immunolabelling of nuclear envelope resident proteins was carried out as described before.^16^ Hereby following antibodies were used: anti-KPNB1 (Abcam; ab2811), anti-Emerin (Novocastra; NCL-EMERIN), anti-Lamin A/C (Santa Cruz; SC-7292), anti-Lamin B1 (Abcam; ab16048), and anti-Matrin-3 (Bethyl; A300-590A). For all antibodies 1:100 dilutions were used.

**REFERENCES**

1. Laurie S, Piscia D, Matalonga L, et al. The RD-connect genome-phenome analysis platform: accelerating diagnosis, research, and gene discovery for rare diseases. *Hum Mutat*. 2022;43(6):717-733. doi:10.1002/humu.24353

2. Benarroch L, Bonne G, Rivier F, Procaccio V, Hamroun D. The 2025 version of the gene table of neuromuscular disorders (nuclear genome). *Neuromuscul Disord*. 2025;46:105261. doi:10.1016/j.nmd.2024.105261

3. Baux D, Van Goethem C, Ardouin O, et al. MobiDetails: online DNA variants interpretation. *Eur J Hum Genet*. 2021;29(2):356-360. doi:10.1038/s41431-020-00755-z

4. Buchan DWA, Jones DT. The PSIPRED Protein Analysis Workbench: 20 years on. *Nucleic Acids Research*. 2019;47(W1):W402-W407. doi:10.1093/nar/gkz297

5. Caputo S, Couprie J, Duband-Goulet I, et al. The Carboxyl-terminal Nucleoplasmic Region of MAN1 Exhibits a DNA Binding Winged Helix Domain. *Journal of Biological Chemistry*. 2006;281(26):18208-18215. doi:10.1074/jbc.m601980200

6. Varadi M, Bertoni D, Magana P, et al. AlphaFold protein structure database in 2024: providing structure coverage for over 214 million protein sequences. *Nucleic Acids Res*. 2024;52(D1):D368-D375. doi:10.1093/nar/gkad1011

7. Ittisoponpisan S, Islam SA, Khanna T, Alhuzimi E, David A, Sternberg MJE. Can predicted protein 3D structures provide reliable insights into whether missense variants are disease associated? *J Mol Biol*. 2019;431(11):2197-2212. doi:10.1016/j.jmb.2019.04.009

8. Rodrigues CHM, Pires DEV, Ascher DB. DynaMut2: assessing changes in stability and flexibility upon single and multiple point missense mutations. *Protein Sci*. 2021;30(1):60-69. doi:10.1002/pro.3942

9. Pauper M, Hentschel A, Tiburcy M, et al. Proteomic profiling towards a better understanding of genetic based muscular diseases: the current picture and a look to the future. *Biomolecules*. 2025;15(1):130. doi:10.3390/biom15010130

10. Klaproth-Andrade D, Scheller IF, Tsitsiridis G, et al. PROTRIDER: protein abundance outlier detection from mass spectrometry-based proteomics data with a conditional autoencoder. Published online February 5, 2025. doi:10.1101/2025.02.01.636024

11. Brechtmann F, Mertes C, Matusevičiūtė A, et al. OUTRIDER: A Statistical Method for Detecting Aberrantly Expressed Genes in RNA Sequencing Data. *The American Journal of Human Genetics*. 2018;103(6):907-917. doi:10.1016/j.ajhg.2018.10.025

12. Benjamini Y, Yekutieli D. The Control of the False Discovery Rate in Multiple Testing under Dependency. *Ann Stat*. 2001;29(4):1165-1188.

13. McLaren W, Gil L, Hunt SE, et al. The ensembl variant effect predictor. *Genome Biol*. 2016;17(1):122. doi:10.1186/s13059-016-0974-4

14. Landrum MJ, Lee JM, Benson M, et al. ClinVar: improving access to variant interpretations and supporting evidence. *Nucleic Acids Res*. 2018;46(D1):D1062-D1067. doi:10.1093/nar/gkx1153

15. Malaichamy S, Idoux R, Polavarapu K, et al. Dominant rhabdomyolysis linked to a recurrent *ATP2A2* variant reducing SERCA2 function in muscle. *Brain*. Published online February 19, 2025. doi:10.1093/brain/awaf067

16. Roos A, Hathazi D, Schara U. Immunofluorescence-Based Analysis of Caveolin-3 in the Diagnostic Management of Neuromuscular Diseases. In: Blouin CM, ed. *Caveolae: Methods and Protocols*. Springer US; 2020:197-216. doi:10.1007/978-1-0716-0732-9_18
