# Supplementary figures and images for: A muscular dystrophy associated with bi‐allelic LEMD2 variants: Expanding the genotype of nuclear envelopathies
Source: Brain Pathol. 2026 Mar 3;36(4):e70082. doi: 10.1111/bpa.70082 (PMC13239970; doi:10.1111/bpa.70082)

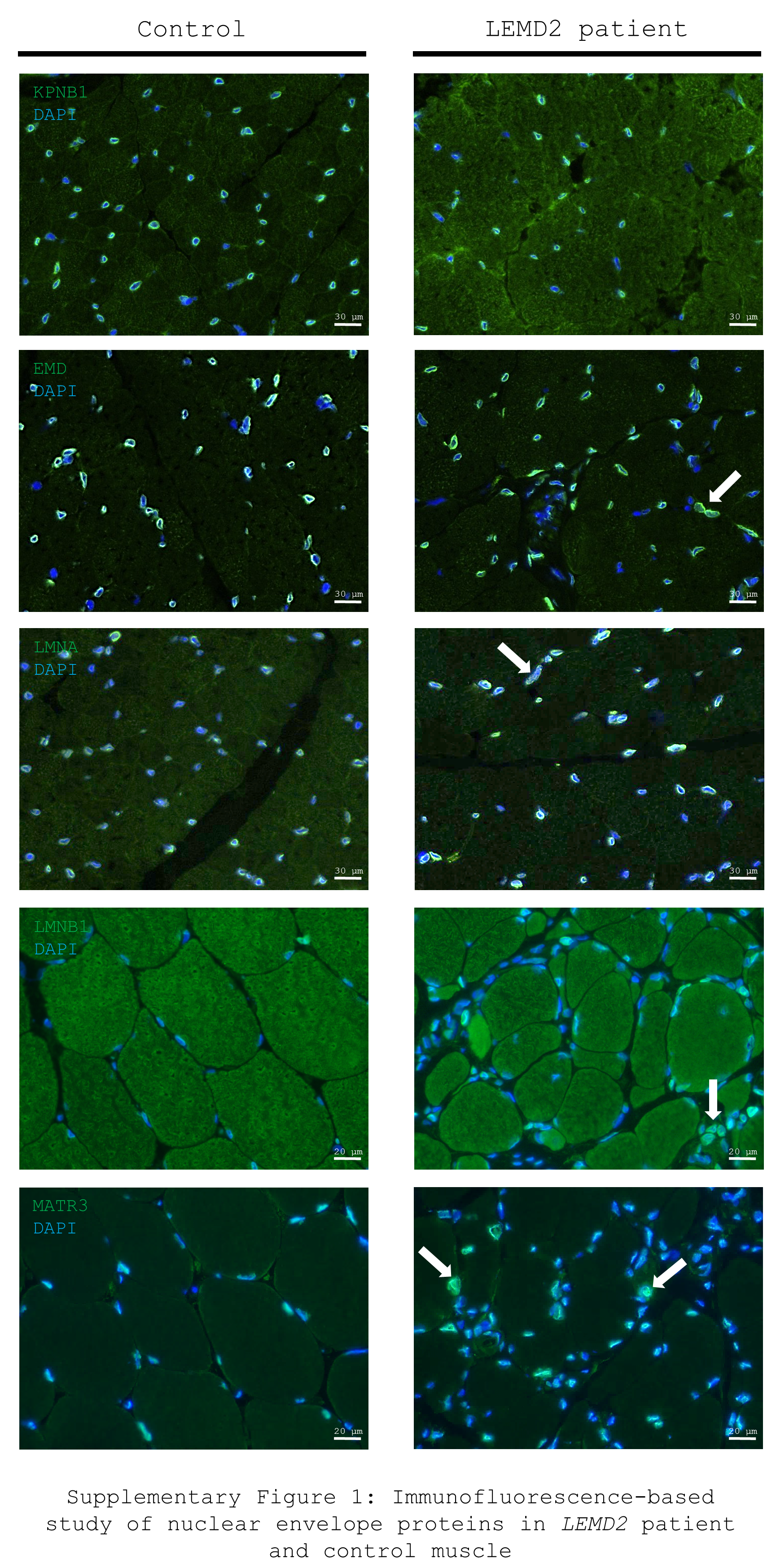

Supplement: Supplementary file 2 — Figure S1. Immunofluorescence‐based study of nuclear envelope proteins in LEMD2 patient and control muscle: Fluorescence‐based immunolabelling of five different nuclear envelope‐related proteins, KPNB1/Importin subunit beta‐1, Emerin, Lamin A/C, Lamin B1 & Matrin‐3 on 7 μm quadriceps biopsy sections of an age matched non‐disease control (left column) and the LEMD2 patient (right column). Altered immunoreactivity for the respective proteins is indicted by white arrows and in sum indicated a pathophysiological impact of the compound heterozygous LEMD2 variants on distribution of other nuclear envelope resident proteins. [file BPA-36-e70082-s002.tif]
